# Evaluation of a pharmacist-led intervention to reduce drug-related problems in patients included in a home healthcare program: study protocol for a pragmatic randomized clinical trial

**DOI:** 10.1186/s12877-024-04763-2

**Published:** 2024-02-19

**Authors:** Clara Salom-Garrigues, Enric Aragonès, Montse Giralt, Cecília Campabadal Prats, Ferran Bejarano-Romero, Laura Canadell

**Affiliations:** 1 Pharmacy Unit, Catalan Health Institute, Joan XXIII University Hospital, Camp de Tarragona Primary Care Area, 4 Doctor Mallafrè Guasch St, 43005 Tarragona, Spain; 2 Research Support Unit, Catalan Health Institute, Camp de Tarragona Primary Care Area, Tarragona, Spain; 3 Mental Health and Primary Care Research Group, 2021 SGR 00989 Tarragona, Spain; 4 Department of Basic Medical Sciences, School of Medicine and Health Sciences, Rovira i Virgili University, Tarragona, Spain; 5 Healthcare Interventions and Community Activities Research Group - GRE ISAC, 2021 SGR 00884 Tarragona, Spain

**Keywords:** Medication review, Pharmacist practice pattern, Primary health care, Domiciliary care, Aged patients

## Abstract

**Background:**

ATDOM is the Catalan home healthcare program at primary care level. Patients in the home care program are usually frail, elderly people with multiple comorbidities. They are often polymedicated, leading to a high risk of drug-related problems (DRPs). Our hypothesis is that the pharmacist-led individualized review of the pharmacotherapeutic plans of ATDOM patients will be effective in improving the quality of treatments by reducing DRPs in terms of indication, adequacy, effectiveness, and safety.

**Methods:**

Aim: To compare the effectiveness of a standardized pharmaceutical intervention for the review and optimization of pharmacological treatments in ATDOM patients with usual management practice.

Design: Pragmatic randomized clinical trial with a comparable control group, with prospective follow-up regarding the intervention on the adequacy of the pharmacological treatment of patients in the ATDOM program.

Setting: Primary care teams in the Camp de Tarragona Primary Care Area, Tarragona, Spain.

Participants: Four hundred and thirty-two ATDOM patients will be recruited, those who are over 65 years old and who are currently undergoing pharmacological treatment.

Measures: Effectiveness of a six-month long intervention in reducing DRPs per patient and polypharmacy. Additionally, in the intervention group we will evaluate the implementation of the proposals for change or improvement made by the responsible physician.

Analysis: The outcomes will be analyzed on an intent-to-treat basis and the analysis units will be the individual patients. Logistic regression and linear regression models will be used to evaluate the effects of the intervention on dichotomous and continuous variables versus the control arm.

Ethics: The protocol was approved by the Research Ethics Committee of the Jordi Gol Primary Care Research Institute (IDIAPJGol), Barcelona, (19/141-P).

**Discussion:**

If the results of the pharmaceutical intervention are favorable, widespread implementation of the program could be possible. It could be extended to all ATDOM patients or outpatients in general. Interdisciplinary teamwork could be strengthened as a result, which would improve the healthcare continuum.

**Trial registration:**

Retrospectively registered. ClinicalTrials.gov Identifier NCT05820945; Registered 21 March, 2023.

## Background

A drug-related problem (DRP) occurs when, in the application and use of drugs, patients experience or are likely to experience problems associated with these drugs [1]. Studies carried out both in and outside Spain show very disparate data on the prevalence of DRPs, with between 3.9% [2] and 59% [3] of patients being affected. This difference in the percentage of DRPs between the different studies could stem from the differing concepts of DRP, in addition to patient typology. The incidence of DRPs is recorded for in-patients [4] and out-patients [5]. This latter group includes those receiving home care. There are a variety of factors which could increase the risk of DRPs in the home care setting: associated illnesses and polymedication (owing to greater life expectancy), sporadic contact with the physician in relation to chronic illnesses which do not require regular check-ups, healthcare via different providers, lack of teamwork by the interdisciplinary team, and scant patient’s information about their treatment [6]. A recent systematic review revealed a high prevalence of DRPs, particularly amongst home-dwelling elderly patients [7]. Certain categories of DRPs, such as adverse reactions to medication or medical interactions, are of considerable clinical significance as they can result in hospitalization [8]. In relation to the importance of DRPs in outpatients, while it is true that the majority of DRPs would not have caused the patient any harm, they could have affected the effectiveness of the treatment [9].

A clinical medication review is a structured process whereby the therapeutic efficacy of each medication is assessed and correlated with the evolution of the pathologies treated. It also studies the prevention and resolution of DRPs, treatment adherence and the understanding the patients have about the pharmacological treatments and their own pathologies. The objective is to decide whether drugs need to be added, withdrawn or continued, and to evaluate the benefits and risks the treatment involves [10]. This is based on the interdisciplinary work of medical professionals, nurses and social workers. The role of pharmaceutical services within the healthcare team must also be considered, with optimal communication between physicians and clinical pharmacists being of particular importance [11]. Intervention led by clinical pharmacists for reviewing medication was able to identify, minimize and resolve DRPs in elderly patients in the primary care services [12] as well as in home care patients [13], especially among the elderly population in home care settings [14].

The Domiciliary Healthcare program (ATDOM), within the Catalan primary care system, organizes care and attends to patients with chronic or acute health problems who have difficulty getting to health centers. The program aims to foster greater autonomy and improve quality of life for patients, and especially to ensure continuity of care, given that it involves the coordination of all healthcare areas [15]. An ATDOM patient's treatment plan is established once the patient and caregiver's needs have been evaluated in terms of such key aspects as communication, coordination, symptom control, continuity of care and treatment support. However, this plan is not proactively reviewed when the patient enters this system or on a periodic basis. Patients such as these, who may be fragile and have multiple morbidities, are more likely to be polymedicated as well.

To optimize medication for polymedicated patients, we can submit these patients' treatment plans to a regular reassessment in order to detect DRPs. In this pharmacotherapy review, the adequacy of the patient's medication plan is assessed and proposals are made with a view to improving the patient's health as well as the economic sustainability of the healthcare system.

This pragmatic study aims to assess the efficacy of the medication review program during the implementation process. Our hypothesis is that the pharmacist-led individualized review of the pharmacotherapeutic plans of ATDOM patients will be effective in improving the quality of treatments by reducing DRPs in terms of indication, adequacy, effectiveness, and safety.

### Methods/design

To design this protocol, the SPIRIT 2013 Statement [16] was addressed. The current version of the protocol is V1, which is registered in ClinicalTrials.gov (ID NCT05820945, first submitted on 21 March, 2023; first posted on 20 April, 2023; see https://www.clinicaltrials.gov/study/NCT05820945 for details). All items from the World Health Organization Trial Registration Data Set are summarized in Table 1.

**Table 1:** World health organization trial registration data set

Data category	Information
Primary registry and trial identifying number	ClinicalTrials.gov Identifier NCT05820945
Date of registration in primary registry	20 April, 2023
Secondary identifying numbers	19/141-P
Source(s) of monetary or material support	Jordi Gol Primary Care Research Institute (IDIAPJGol)
Primary sponsor	Fundació d'Investigació en Atenció Primària Jordi Gol i Gurina
Secondary sponsor(s)	Catalan Health Institute
Contact for public queries	CS [csalom.tgn.ics@gencat.cat]
Contact for scientific queries	CS, Pharmacy Unit, Camp de Tarragona Primary Care Area, Catalan Health Institute, Tarragona, Spain
Public title	A Pharmaceutical Intervention to Reduce Drug-Related Problems in a Home Healthcare Program
Scientific title	Evaluation of a Pharmacist-Led Intervention to Reduce Drug-Related Problems in Patients Included in a Home Healthcare Program: A Pragmatic Randomized Clinical Trial
Countries of recruitment	Spain
Health condition(s) or problem(s) studied	Polypharmacy Potentially Inappropriate Medications
Intervention(s)	Active comparator: pharmacist-led medication review at patient level, and change proposals at physician level
	Placebo comparator: usual pharmacotherapy management
Key inclusion and exclusion criteria	Inclusion Criteria: patient in the home care program, sixty-five years of age or older, and active pharmacological treatment plan with at least one drug
	Exclusion Criteria: the responsible physician considers that participation may harm the patient
Study type	Interventional
	Allocation: randomized; Intervention model: parallel assignment; Masking: single (outcomes assessor)
Date of first enrolment	2 March, 2020
Target sample size	432 patients
Recruitment status	Recruiting
Primary outcome(s)	Total DRPs, DRPs per patient, and number of patients with one or more DRP (time frame: baseline and 6 months follow-up) Number of associated drugs per patient and number of polymedicated patients (≥ 8 different drugs, time frame: baseline and 6 months follow-up)
Key secondary outcomes	Number of proposals issued by the pharmacist (time frame: baseline and 6 months follow-up) Number of proposals implemented by the physician (time frame: 6 months follow-up)

*DRP *Drug-related problem

### Aim

The aim is to compare the effectiveness of a standardized pharmaceutical intervention for the review and optimization of pharmacological treatments in elderly patients on the ATDOM program with usual management practice.

In particular we want to determine the effectiveness of the program by way of a six-month intervention trial in order to (1) reduce DRPs per patient, and (2) reduce polymedication. In the intervention group, we will evaluate the implementation of proposals for change or improvements made by the physician in charge.

### Design

Pragmatic controlled clinical trial for assessing healthcare intervention with random distribution of patients into two groups: (a) intervention group, in which patients will receive the pharmaceutical intervention being assessed, and (b) the control group, in which the patients' pharmacotherapy plans will be managed according to the usual criteria and procedures. Pharmaceutical intervention forms part of a range of primary care services and its assessment will be carried out under implementation conditions in real healthcare practice. If this pharmaceutical intervention – which is the object of assessment – proves effective, the patients in the control group will receive the same treatment once their participation in the study is over.

### Study setting and patient sample

The study will be carried out at 10 primary care centers within the Camp de Tarragona Regional Health Authority (Catalan Health Institute) in the Tarragona province (Catalonia). Participation in the study by family physicians from these centers will be voluntary.

The patients from the participating physicians will be eligible if they receive care through the ATDOM program, if they are 65 years old or over, and if they have an active pharmacological treatment plan involving at least one drug. It will be up to the responsible physician to decide if there are any reasons why a patient may be excluded, for example, if their participation might cause them harm. Those patients who are taken into care homes or long-term nursing facilities, or who pass away during the period of the study will also be excluded from the analysis.

### Sampling and randomization of patients

The eligible patients will be assigned by a primary care pharmacist either to the intervention group or the control group from the patient lists of each participating physician, following a random procedure using the https://www.random.org/ platform.

### Intervention

The intervention will consist of a primary care pharmacist carrying out a clinical review of the medical records and pharmacological treatment plans established for the patients registered on the ATDOM program. The pharmaceutical review procedures are systematized in the document "Rational Use of Medication. Basic medication management in chronic patients: reconciliation, review, deprescribing and adherence” [17], published by the Catalan Department of Health, part of the Camp de Tarragona Regional Authority’s primary care services portfolio (Catalan Health Institute). The pharmacist will review the instructions for each drug in the treatment plan in terms of the patient's pathologies, how appropriate the medication is for their age and clinical conditions (e.g. kidney failure, liver failure or other comorbidities), the suitability of the dosage, treatment instructions and duration, the effectiveness of the treatment in relation to the intended therapeutic goal, the safety of the medication and adherence to the treatment (Table 2). This review will be based on the Beers 2019 criteria [18], STOPP-START [19], Priscus [20] and EU(7)-PIM list [21]. Adherence to the treatment will be monitored using information about the medicines dispensed from the pharmacy office which is registered on the electronic prescription module.

**Table 2:** Classification of drug-related problems (DRPs) that can be detected in a pharmaceutical review

Type	Drug-related problem	Example
**Indication**	Unnecessary medicine	An antiplatelet drug used preventatively in primary care is an unnecessary medicine according to current guidelines
	Medicine not indicated or inappropriate for the condition it is intended to treat	
	Lack of prescription for a necessary medicine	
**Adequacy**	Inappropriate medicine for the patient owing to age, medical situation or underlying pathology	Glibenclamide is an inappropriate medicine in geriatrics as it is a sulfonylurea with a prolonged half-life which can cause serious hypoglycemia
	Dose is higher or lower than the correct/recommended dose	
	Frequency of administration incorrect or not recommended	
	Drug form incorrect or not recommended	
	Treatment is longer or shorter than the correct or recommended period	
	Administration time incorrect or not recommended	
**Effectiveness—efficiency**	Lack of adherence	A drug is not effective for a patient because he/she is not taking it
	Therapeutic goal is not reached	
	Therapeutic goal is reached but the intensity of the pharmacological treatment must be reduced	
	Not the most effective-efficient alternative	
**Safety**	History of allergy or other adverse reaction to the same medication or similar	Metformin is a drug which is not recommended for patients with glomerular filtration of < 30 ml/min/1.73 m^2^)
	Drug with contraindications	
	Therapeutic duplication	
	Drug-drug interaction	
	Drug-food interaction	
	Lack of analytical control	

**Fig. 1:**
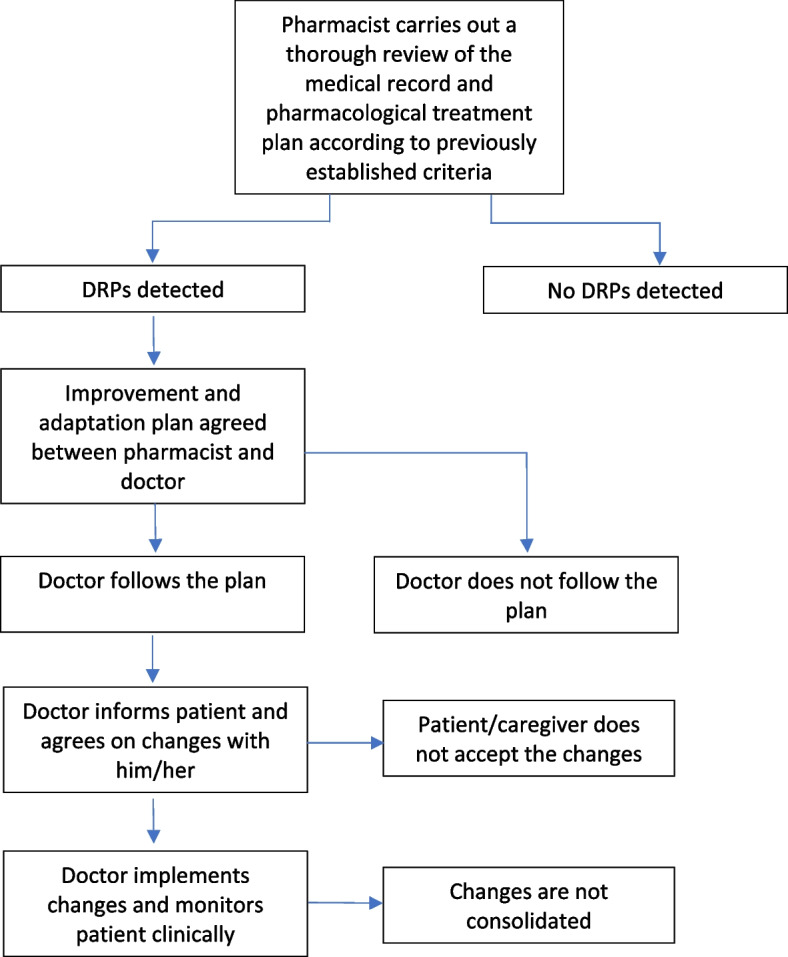
Flow diagram of the pharmaceutical review intervention and improvements to the medication plan

After the review process, a meeting will be held between the pharmacist and the physician in charge in which the DRPs detected will be discussed (Fig. 1) and a series of recommendations for improvement and proposals for change will be made. The proposals might be: (a) suspending treatment of a drug, (b) changing the medication, (c) changing the dosage, (d) changing of posology, (e) starting a new treatment, (f) monitoring the clinical and/or analytical variables, and (g) interventions to encourage adherence to the treatment. Thereafter, the physician will make a global assessment of the improvement and adaptation treatment plan and in exercising his/her responsibility will decide to what degree and/or which points to adhere to in the plan. In any case, the proposals, their justification, the expected advantages and possible drawbacks will be explained and agreed with the patient and, if necessary, with the caregiver or responsible family member. The whole procedure will be recorded in the patient's clinical record.

Although the intervention being assessed will be carried out both on a professional level with the family physicians as well as with the patients and caregivers, the intervention results will be measured in every patient. The intervention on physicians/patients will not be blinded but the results will be measured blind.

### Usual practice (control group)

In the control group, the patients' treatment plans will be managed according to usual criteria and procedures, including an automatic warning system generated by the Self-Audit tool which is part of the electronic clinical record to identify safety problems: redundant treatments, polymedicated patients, side effects, and review of the treatment duration, among others [22].

### Measurements

The outcome variables will be obtained at baseline and after six months with a review of the pharmacological prescription module in the electronic clinical record made using a questionnaire which compiles electronic data. The review will not be blind with regards to the patient's allocation to either of the two study groups. However, the results will be subsequently evaluated blind. A data monitoring committee (DMC) is not needed in this monocentric academic trial with known minimal risks. All study-related information will be codified and stored securely at the study site.

### Variables

#### Principal variables

- Drug related problems [23, 24]: Total DRPs, DRPs per patient, and number of patients with one or more DRP.
- Polymedication: number of associated drugs per patient and number of polymedicated patients. We define polymedication as the simultaneous consumption of ≥ 8 different drugs [25].

#### Secondary variables and co-variables

Sociodemographic data: age, sex, primary care team, socioeconomic status according to place of residence [26], household characteristics, caregiver, social/family support.

Clinical variables:

-  Physical comorbidity: chronic pathologies such as diabetes mellitus, coronary heart disease and other types of heart disease, cerebrovascular disease, respiratory diseases, musculoskeletal diseases, diseases of the connective tissue and neoplasms.
- Psychological comorbidity: dementia, depression, anxiety and psychotic disorder.
- Charlson index. This refers to a global index of comorbidity which computes 19 items related to various pathologies which together with the patient's age is used to predict comorbidity after one year and after ten years [27].
- AMG (Adjusted Morbidity Groups) is an instrument for stratifying the population in relation to multimorbidity and complexity. It provides good explanatory results for indicators of healthcare resource usage, in particular for drug consumption [28].
- Barthel test. This measures the degree of the patient's dependence in basic day-to-day activities and its results are shown on an ordinal scale from 0 (maximum dependence) to 100 (total autonomy) [29, 30].
- Pfeiffer test. This questionnaire detects the existence and extent of cognitive decline by assessing the subject's responses to ten brief questions. It examines short- and long-term memory, orientation, information about daily life and calculation ability [31, 32].
- Complex chronic patient (CCP). This is defined as a patient with multimorbidity, extreme fragility or a unique condition which makes clinical management difficult. These are patients who use emergency hospital services very frequently, spending various periods in hospital throughout the year, they experience reductions in their personal autonomy either temporarily or permanently and are polymedicated [33].
- Advanced chronic patient (ACP). These are defined as patients with a limited life prognosis and high level of need, requiring palliative care and advanced decision planning [34].

Variables related to pharmacological treatment:

- Description of the pharmacotherapy plan using an ATC code (Anatomical, Therapeutic, Chemical classification system). This is a drug index organized by therapeutic group, which includes the system or organ which it acts upon, the pharmacological effect, the therapeutic indications and the chemical structure of the drug [35].
- Description of DRPs detected according to typology: indication, adequacy, effectiveness, safety (Table 2) [8].
- Adherence to the treatment by analyzing the medicines dispensed from the pharmacy office in relation to the dosage and prescribed dose. This will be examined after a period of six months. The adherence will be considered adequate if > 80% of the necessary doses have been taken.

Variables related to the procedure and result of the pharmaceutical intervention:

- Number of changes or improvement proposals sent by the pharmacist.
- Number of proposals implemented by the physician.

### Statistical methods

#### Sample size

**Fig. 2:**
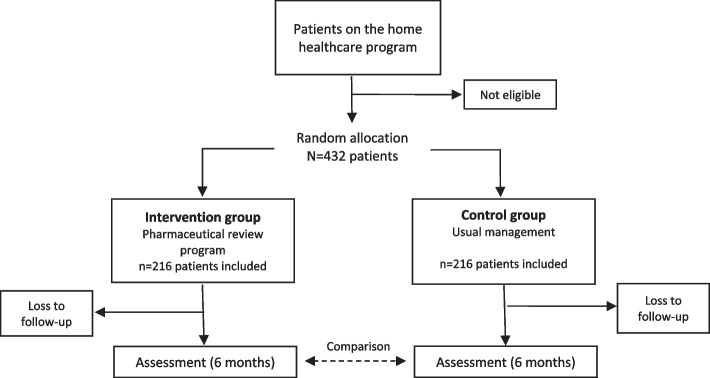
Flow diagram of study: center allocation, sampling and monitoring

Based on a review of the literature [7, 14], as well as an exploratory analysis of 100 patients eligible for the study, we assume that in our target patient population the proportion of patients with at least one DRP will be around 60% and we hope to be able to detect a reduction of ≥ 15% in the intervention group after six months. If we accept an alpha risk of 0.05 and a power of 80% in a bilateral contrast, and assume a 20% loss of patients in the process (patients who go into a care home, or into a long-term nursing facility, or who pass away during the study period), we need to include 216 individuals in each group (Fig. 2).

### Data analysis

The principal analyses will be carried out by intention to treat, taking the patients in each group according to their initial random assignment, independently from the adherence to the pharmaceutical intervention and the recommendations made. The results will be measured in individual patients, although the intervention will be largely directed to the attending physician in the form of instructions or change recommendations. In the analyses we will bear in mind the potential loss of independence that might occur in observations when patients are treated by the same physician (cluster effect). We will first compare both study groups to check that their baseline characteristics are similar. The principal result variables (dependent) will be the number of DRPs per patient, the proportion of patients with DRPs, the number of drugs per patient and the proportion of polymedicated patients. To assess the effects of the intervention on the dichotomous variables we will use multilevel logistic regression models with mixed effects using an Odds Ratio estimate (IC95%) of the intervention group with respect to the control group as an effect measure. To measure the effect on the continuous variables we will use linear regression models with random effect, estimating the differences in adjusted means (IC95%) for the intervention group versus the control group. The statistical significance will be established as *p* < 0.05. For these calculations, we will use the R statistical package.

## Discussion

There is a high incidence of DRPs in elderly patients, including those in home care [6, 7]. These DRPs often involve morbimortality and have a high economic cost, which can even exceed the cost of the prescribed medicines [36]. Pharmaceutical intervention by way of a medication review could reduce these DRPs, which cause unnecessary suffering to patients and carry a high social cost. Furthermore, the potential cost saving in primary care could far exceed the cost in human resources currently being invested in identifying and dealing with DRPs [37].

The Catalan primary care system has a program called ATDOM which complies with the directives of the Program for the Prevention and Care of Chronicity, instigated by the Catalan Government’s Department of Health [33], which since March 2019 includes a prescribed medicine review program. The aims of this study fit the Catalan health authorities' strategy to strengthen and improve chronic care at home. The present pragmatic study aims to assess the efficacy of the medication review program during the implementation process.

The efficacy of pharmaceutical intervention for the review of medication, identification and resolution of DRPs in elderly patients has been assessed in hospitals [38] and in care homes [39], as well as in primary care [12]. One descriptive study showed a greater number of DRPs in elderly patients in home care than in elderly residents of care homes [40]. The effect of medication reviews for home-dwelling elderly patients has been studied at an observational level [41], including patients receiving home care [42]. However, we only found a randomized comparative study in home-dwelling older people referred to an aged care assessment team [43]. The present study is experimental, designed as a controlled randomized clinical trial, which will assess the effectiveness of standard pharmaceutical intervention for reviewing and optimizing drug treatment in elderly patients on the ATDOM program, with respect to the usual management practice.

The experimental design of this study – a controlled randomized clinical trial – has the potential to give results with a higher level of clinical evidence than observational studies [44]. Being a pragmatic study, the inclusion criteria are broader and the sample is more heterogeneous so it may have lower internal validity. However, the results will be more similar to the real-life conditions of home care for elderly patients and therefore more easily replicable, not only for the institution where the study was carried out, but also more generally in similar fields throughout the healthcare system.

Standard pharmaceutical intervention for the review and optimization of drug treatments in elderly patients could be extended to all ATDOM patients or even outpatients in general, with health benefits for the patient and economic sustainability for the healthcare system. In this way, good medical practice will be improved by incorporating pharmacotherapy instructions based on the best scientific evidence available. This will make it possible to strengthen interdisciplinary teamwork, supporting the continuum of care, especially in pharmacist-physician communication, where criteria are not always shared in terms of prescription, review of medication and medicine usage. Finally, this clinical trial should be complemented with a study of the cost–benefit relationship which could inform healthcare planning managers in their decision-making.

## Data Availability

The datasets used and/or analyzed during the current study will be available from the corresponding author on reasonable request. The study results will be released to the participating physicians, patients and the general medical community. The International Committee of Medical Journal Editors authorship criteria will be addressed in manuscripts submitted for publication.

## Abbreviations

ATC: Anatomical Therapeutic Chemical classification System
CEIC: Clinical Research Ethics Committee
DRPs: Drug-related problems
AMG: Adjusted morbidity groups
IDIAPJGol: Jordi Gol Primary Care Research Institute
ACP: Advanced chronic patient
CCP: Complex chronic patient
